# Neoantigen evolution in head and neck cancer progression: where do we go from here?

**DOI:** 10.18632/oncotarget.27942

**Published:** 2021-06-08

**Authors:** Ari Joseph Rosenberg, Evgeny Izumchenko

**Keywords:** head and neck cancer, immunotherapy, neoantigens

Locoregional head and neck squamous cell carcinoma (HNSCC) can be treated with multimodality therapy, however approximately 50% of patients will develop recurrent disease either locally or distantly [1]. While in select settings recurrent disease can be treated with curative salvage therapy, many recurrences are treated palliatively. Systemic therapies in this setting include cytotoxic chemotherapy, targeted therapy, and immunotherapy [2]. Recently, anti-programmed death 1 (PD-1) monoclonal antibodies have demonstrated a survival benefit in recurrent/metastatic (R/M) HNSCC either as monotherapy or in combination with chemotherapy [3–5]. However, this survival benefit is driven by a small subset of R/M HNSCC patients that respond to this therapeutic strategy, as primary and secondary resistance represent substantial barriers to harnessing anti-tumor immunity in many patients. Elucidating tumor-specific neoantigens through the progression of HNSCC from primary to R/M settings is critical to developing novel systemic immunotherapeutic strategies to improve patient survival in this disease.

Van Tine and colleagues set out to characterize genomic and neoantigen evolution from primary tumor to R/M HNSCC [6]. The investigators evaluated twenty-three paired primary and recurrent HNSCC tumors, and performed whole exosome sequencing, RNA-seq, and immunohistochemistry to characterize genomic and neoantigen evolution in the setting of disease progression. A heterogeneous cohort was represented with a mix of anatomic primary sites (oropharynx, larynx, hypopharynx, and oral cavity), human papillomavirus-associated (HPV+) and HPV-negative cases, and various treatment modalities including surgical resection, radiotherapy, and chemotherapy. Genomic characterization of somatic mutations demonstrated a dynamic increase in number of recurrently mutated genes, however the mutational landscape was highly heterogeneous, with only a few patients demonstrating shared mutational events in matched primary and R/M tumors. While the spectrum of T cell infiltration was no different, programmed death ligand 1 (PD-L1) expression was increased in the R/M tumors, whereas levels of antigen presenting genes were significantly decreased. Neoantigen discovery among primary and R/M tumors revealed that patients with a larger number of somatic mutations had higher neoantigen burden. The majority of predicted neoantigens were unique to an individual’s tumor, while only a small number of patients shared revolving events, albeit with a trend towards improved survival and higher CD3+ CD8+ T-cell infiltration. According to the authors, these observations are suggestive that specific neoantigen presentation may drive antitumor immunity and contribute to patient survival outcomes.

The role of immunotherapy in the treatment paradigm of HNSCC continues to evolve. Nivolumab, an anti-PD-1 monoclonal antibody, was evaluated in a randomized phase 3 trial of platinum-refractory R/M HNSCC in which nivolumab demonstrated one-year overall survival of 34.0% compared with 19.7% for investigator’s choice chemotherapy (HR 0.71, 05% CI 0.55–0.90) [3]. Another anti-PD-1 monoclonal antibody, pembrolizumab, was evaluated in a similar patient population of R/M HNSCC who failed platinum therapy randomized to pembrolizumab or investigator’s choice, and demonstrated an improvement in one-year survival of 37% versus 27% respectively (HR 0.80, 95% CI 0.65–0.98) [4]. The recent Keynote-048 trial demonstrated that pembrolizumab alone in patients with PD-L1 combined positive score (CPS) of ≥ 1 and pembrolizumab with chemotherapy regardless of CPS is associated with an overall survival benefit. These observations established a new standard of care, incorporating immune checkpoint blockade in the front-line R/M setting for HNSCC [5]. Although a subset of patients with R/M HNSCC benefit from this strategy, a majority of patients have disease that fails to respond and requires an alternative approach to overcomes mechanisms of tumor immune evasion. While assessment of PD-L1 expression by immunohistochemistry is an important biomarker that enriches for responders, improved biomarkers for therapeutic benefit and a deeper mechanistic understanding is critical to advancing the field beyond immune checkpoint blockade in the R/M setting. Van Tine and colleagues [6] performed a small exploratory study of matched primary and recurrent tumors to contribute to mechanistic insights into the role that evolving genomic alterations and associated neoantigens play in the antitumor immune response. While the small size, heterogeneous patients population, and exploratory nature of this study limits our ability to derive definitive conclusions, the observation of shared neoantigens in the context of a trends towards increased CD8+ T-cell infiltration, cytolytic activity, and improved survival outcomes, can serve as hypothesis generating regarding the potential role some neoantigens play in the anti-tumor immune response. Nevertheless, prospective genomic and neo-antigenic characterization along the course of disease in the context of immune checkpoint inhibitor therapy is needed to support these suggestions.

Despite the impressive survival benefit of immunotherapy in the R/M HNSCC setting, this treatment modality has thus far failed to improve outcomes in the curative setting accentuated by the negative results of two randomized trials that combined anti-PD(L)1 therapy concurrently with definitive chemoradiation [7, 8]. This raises important questions into how immunotherapy can optimally be incorporated into the current multimodal approach in locoregional HNSCC [9]. Some considerations with moving immune checkpoint blockade forward into the curative setting include the timing of immunotherapy, standard fractionated radiation with large volume lymph node irradiation, and concurrent systemic therapies. Results reported by Van Tine and colleagues highlights the genomic and neo-antigenic evolution from primary to R/M HNSCC as an area that requires additional investigation into why the survival benefit of immune checkpoint blockade in patients with R/M disease has yet to be translated into the early stage/curative setting.

In conclusion, the authors should be commended for their work in this small exploratory study, as we strategically build on the success thus far in harnessing the immune system against head and neck cancer, further mechanistic discovery into tumor-associated neoantigens that drive response will be critical to moving the field forward.
